# Remote access protocols for Desktop-as-a-Service solutions

**DOI:** 10.1371/journal.pone.0207512

**Published:** 2019-01-04

**Authors:** Eduardo Magaña, Iris Sesma, Daniel Morató, Mikel Izal

**Affiliations:** 1 Public University of Navarre, Department of Electrical, Electronic and Communications Engineering, Pamplona, Spain; 2 Institute of Smart Cities, Pamplona, Spain; University of Nevada, UNITED STATES

## Abstract

The use of remote desktop services on virtualized machines is a general trend to reduce the cost of desktop seats. Instead of assigning a physical machine with its operating system and software to each user, it is considerably easier to manage a light client machine that connects to a server where the instance of the user’s desktop machine actually executes. Citrix and VMware have been major suppliers of these systems in private clouds. Desktop-as-a-Service solutions such as Amazon WorkSpaces offer a similar functionality, yet in a public cloud environment. In this paper, we review the main offerings of remote desktop protocols for a cloud deployment. We evaluate the necessary network resources using a traffic model based on self-similar processes. We also evaluate the quality of experience perceived by the user, in terms of image quality and interactivity, providing values of Mean Opinion Score (MOS). The results confirm that the type of application running on the remote servers and the mix of users must be considered to determine the bandwidth requirements. Applications such as web browsing result in unexpectedly high traffic rates and long bursts, more than the case of desktop video playing, because the on-page animations are rendered on the server.

## Introduction

Traditional desktop computers executing local productivity applications are evolving into light local computers used as remote displays for centralized machines. This is the scenario of Remote Desktop (RD) systems [1], where a host streams a computer desktop environment to the user’s machine, where the user then browses this desktop as if it were local. This is a common deployment scenario in large and medium size enterprises owing to the reduction in capital and operational expenditures it provides [2].

The centralized computing resources are typically virtualized. A single host can offer independent desktops to dozens of users in what is called a Virtual Desktop Infrastructure (VDI) [3]. These remote desktops are accessed from thin clients: computers with reduced computational power and small disks that are used solely as remote displays and input devices (keyboard, mouse, sound, USB ports). The user employs a desktop operating system that behaves as if local to his computer, yet is instead a network-streamed version of a virtualized and centralized desktop.

RD systems provide reduced computer management costs owing to centralized backups, updates, security control, and other system functions. The capital expenditure in desktop hardware is also reduced as it functions with inexpensive thin clients. New features can be easily offered, such as access to the user’s desktop from any device, even mobile devices (from smartphones or tablets). The main disadvantages to these RD systems are the requirement for a centralized infrastructure that offers the real desktop and the requirement of high speed and low latency network access from the users to the infrastructure.

The centralized infrastructure in a VDI is typically a private server farm, datacentre, or private cloud, for the exclusive use of the company employees. However, a VDI is complex and hence is difficult to manage and maintain. The consequence of these challenges has been the increase of Virtual Desktop Clouds (VDCs) and Desktop-as-a-Service (DaaS) solutions. They offer the capability to outsource the infrastructure and its management to a public cloud. However, VDC usability imposes more severe requirements on the minimum network speed and maximum latency for the company Internet access links and the path from the local network to the closest DaaS cloud [4]. Some providers even recommend dedicated links to their cloud [5]. The minimum required bandwidth and maximum delay for a population of VDC users depends on the specific RD system used, because of the different compression algorithms, protocol behaviours, and user profiles [6].

There is a significant diversity in RD solutions. Remote FrameBuffer protocol (RFB) [7] and Remote Desktop Protocol (RDP) [8] are two of the oldest RD protocols. They are implemented in commercial and open source packages for the majority of operating systems, from any X Window system to Android, macOS, and the different Microsoft Windows versions. Virtual Network Computing (VNC) is a desktop sharing solution that implements RFB. RDP is included in Windows operating systems. Another popular multi-platform RD solution used for controlling remote physical hosts is TeamViewer [9]. However, TeamViewer is not designed for a VDI environment; rather, it is used for remote assistance support [10].

PCoIP [11] is a protocol used in several RD systems (for example, VMware View [12]). It is particularly popular since its adoption for Amazon WorkSpaces in 2015 [13], which is a DaaS that offers remote desktop seats according to requirements, with pay-per-use plans and no requirement to administer the management of the server infrastructure. This new proposal from Amazon opens an interesting question on the evaluation of the different solutions of VDI, both at the level of network resources required and the quality of experience (QoE) perceived by the users.

Owing to the popularity of Microsoft Windows as a desktop operating system, large RD deployments are based on Windows desktops and use RD services optimised for Windows. Citrix is based on the Independent Computing Architecture (ICA) protocol and represents more than 50% market share within the RD space, mainly for Windows desktops [2]. It is followed by VMware (that uses PCoIP as the protocol), with approximately a 30% share, and RDP with less than a 5%. Other offerings such as Dell/Quest vWorkspace, Virtual Bridges, Oracle/Sun VDI, and Red Hat Enterprise Virtualization have less than a 5% market share.

In this paper, we compare the most important Remote Desktop protocols and software implementations, including the effects of WAN access and public cloud sharing for a DaaS scenario. We deploy the setups in the Amazon cloud environment (Amazon Web Services, AWS). We present a novel comparison of the most representative Remote Desktop protocols using virtualized hosts in an Amazon datacentre accessed by clients from a campus LAN. We deploy server software for ICA HDX (used in VDI Citrix systems [14]), RFB (VNC), RDP (used in Windows Azure [15]), and TeamViewer protocols, and compare these to the PCoIP protocol as provided by Amazon WorkSpaces [16] (the DaaS provided by Amazon). We define three user profiles: the first centred on an office productivity suite, the second based on Internet browsing, and finally a multimedia video consumer. We compare video quality, interactivity, and link bandwidth requirements for the aforementioned protocols. We also model the network traffic they produce using long-range dependent processes. This model is used to evaluate the different degree of burstiness due to the different user profiles or characteristics in the remote desktop protocols.

The results demonstrate the trade-offs among the different RD systems, between quality and bandwidth consumption. The different approaches in system design result in specific protocols being more adequate for specific desktop user tasks. For example, the Amazon WorkSpaces solution (based on PCoIP) presents a reasonable quality, however its network bandwidth usage is high. Conversely, TeamViewer sacrifices video quality and interactivity to contain network usage.

This paper is organised as follows. The next section compares this work with previous related papers in the literature. The section “Methodology and experimental setup” describes the experimental environment including the hardware and software installations, user profiles, and evaluation metrics. Section “Evaluation of PCoIP” presents the measurements and performance results for the PCoIP protocol (Amazon WorkSpaces DaaS), followed by a section that compares the results from PCoIP to the other RD protocols. The section “Recommendations for remote desktop protocol design” provides suggestions to improve the user experience in these scenarios. Finally, conclusions are presented.

## Related work

There are works in the literature that compare RD protocols using different metrics, however none of these offer traffic models or QoE measurements with virtualized hosts in a public cloud. In [17] the authors present a comparison of RD solutions in a WAN environment, focusing on latency, bandwidth, and video quality. The quality is estimated from the amount of traffic in the network, without evaluating the real experience perceived by the user. Further, PCoIP was not popular when this research was conducted, therefore it was not included in the comparison. In [18] they include PCoIP in a virtualized environment and emulate a WAN scenario adding delay and losses to the traffic. However, in both [17] and [18] the authors evaluate video quality based on the transfer size, where a smaller size is assumed to be related to a lower quality. Conversely, we compare the video stream at the source and destination and from a Peak Signal-to-Noise Ratio (PSNR) measurement, we conclude a Mean Opinion Score (MOS) value. The MOS is a popular metric for QoE in video streaming scenarios [19].

Suzbjevic *et al.* [20] conducted QoE measurements for a population of RDP users working with different applications (document editing, audio and video streaming, and web browsing). In [21] the authors conducted a similar QoE study, however for the ICA protocol. Although both papers provide MOS values, they focus exclusively on a single RD protocol, whereas in this paper we compare RDP to VNC, ICA, and particularly PCoIP in the Amazon WorkSpaces cloud.

In [22] Schlosser *et al.* evaluated QoE by measuring the time to complete a user task (typing, scrolling). They were interested in the effects of several optimisation mechanisms in Citrix ICA. They did not compare to other RD solutions and did not evaluate the change in quality in the video stream. In a previous work [23], the same authors did compare RDP to ICA; however, they used the same QoE metrics and did not include PCoIP in any of the cases.

More recent papers have compared PCoIP to RDP [24] [25]; however, they neither offer QoE measurements nor perform the experiments in a real cloud environment. Further, in the majority of the above mentioned papers, the evaluation only included the effect of network losses and delay in an emulated environment based on tools such as NetEm [21].

## Methodology and experimental setup

In this paper, we compare the remote desktop protocols RDP, ICA, PCoIP, RFB, and TeamViewer. RDP is used in Microsoft Azure VDI [15], Amazon EC2 [26], and VMware [27]. ICA is the protocol used in Citrix VDI [14]. PCoIP is used in VDI systems such as Amazon WorkSpaces [16] and VMware [12]. RFB is the protocol under VNC [7].

In this section, we first highlight the characteristics in each protocol that are relevant for this comparison. We then present the hardware and software tools used for the evaluation, describe the user profiles, define the measurement metrics, and finally introduce the characteristics of self-similar arrival processes that are used for user traffic modelling.

### Protocols for remote desktop

ICA (1989) is a proprietary solution by Citrix Systems. It was developed for remote access to Microsoft Windows desktops; today, it also offers access to Linux hosts. It uses mainly TCP as a transport protocol [28], however audio streams can be sent using UDP [29]. It transports graphic interface function calls. It offers different priorities for different flows and it transports originally compressed multimedia streams employing separate virtual communication channels [30] [31].

VNC (1998) implements the RFB protocol, specified today in [7]. There exist client and server implementations for the majority of operating systems including Microsoft Windows, Linux, macOS, and Android. As an open standard, there are commercial and free software packages such as RealVNC [32], TightVNC [33], UltraVNC [34], and TurboVNC [35]. As its name implies (Remote FrameBuffer protocol), it functions at the framebuffer level, capturing the stream sent to the video output. Using TCP, the client asks the server for updates on different parts of the screen [7] [36]. The reply can be raw pixmaps (or other content such as audio) with different types of compression and quality depending on network conditions.

RDP (1998) is a protocol developed by Microsoft for Windows systems based on the T.120 family of protocols from the International Telecommunication Union, with third party client and server implementations for other platforms (including Linux, macOS, and Android). It uses TCP as the transport protocol and supports multiple parallel channels for the transmission of different flows of data [8]. It reduces the amount of traffic by transporting graphic interface function calls instead of bitmaps when possible. Originally compressed multimedia streams can be redirected from the server to the client for local client playback (without decompressing in the server), reducing CPU load on the server and network traffic [37].

TeamViewer (2005) uses its own intermediate servers to provide the service in a manner that facilitates the connection with remote desktop servers that are behind a Network Address Translator (NAT). Authentication is performed from the client and remote desktop server against the TeamViewer servers. When the identity is verified, the intermediate servers control connecting the client to the remote desktop server. The control communication is implemented over TCP [38] and the actual communication of the remote desktop service with the server uses UDP. The screen is updated as a pixel map in a similar manner to VNC, however in this case, over the UDP transport protocol. The type of information to be displayed on the screen is automatically detected and compressed accordingly, considering also the network conditions. Initially the different areas of the screen are loaded with low resolution; in the event that the content requires higher resolution (high quality images or videos), it is resent with a higher quality layer.

The PCoIP (2008) protocol was developed by Teradici Corporation. It is today offered by VMware View installations [12] and the Amazon WorkSpaces virtual desktop cloud [16]. There are Windows and Linux installers available for the server and client for the majority of platforms including Android and iOS tablets. PCoIP streams an on-the-fly compressed video from the screen output, using dynamic compression based on the type of content (text, image, video). It transports the compressed pixmap data over UDP [39].

The characteristics of these RD protocols are summarised in Table 1.

**Table 1:** Characteristics of RD protocols.

RD protocols	VNC	RDP	ICA	TeamViewer	PCoIP
VDI using protocol	-	Windows Azure Amazon EC2 VMware	Citrix	-	WorkSpaces VMware
Transport protocol/ports	TCP/5900	TCP/3389	TCP/1494, 2598	TCP/5938, 80, 445 UDP/dynam.	UDP/4172
Update submission strategy	pixel map	graphic interface function calls	graphic interface function calls	pixel map	pixel map
Compression adapted to network conditions	no	yes	yes	yes	yes
Compression adapted to content	no	yes	yes	yes	yes
Screen refresh controlled by	client	server	server	server	server
Server operating system	Windows macOS Linux	Windows Linux	Windows Linux	Windows macOS Linux	Windows
Client operating system	Windows macOS Linux iOS Android	Windows macOS Linux iOS Android	Windows macOS Linux iOS Android Blackberry	Windows macOS Linux Chrome OS iOS Android Blackberry	Windows macOS Chrome OS iOS Android Fire
Open source	yes (depending on implementation)	no	no	no	no
Computing resources at server	medium	low	medium	medium-low	medium-high
Computing resources at client	medium-high	medium-low	low	medium	very low

### Hardware and software infrastructure

The scenario of remote desktop solutions in a real cloud environment, accessed from users through the public Internet, has not been studied in the literature. To address this deficiency, we deploy different remote desktop servers in the Amazon public cloud (Amazon EC2). We selected the Amazon datacentre in Ireland owing to its close location to our remote desktop clients in Spain.

We created two similar virtual servers in the cloud. For the PCoIP evaluation, we selected the “value package” template in Amazon WorkSpaces. This is a Windows 7 installation on one CPU core running at 2.4 GHz and using 2 GB of RAM. The second virtual server was created to evaluate RDP, RFB, and TeamViewer. To achieve a fair comparison, we selected the most similar offering in the same datacentre. It was an EC2 instance using Windows Server 2012 on one CPU core at 2.4 GHz and 2 GB of RAM. On this virtual server, we evaluated RDP8 (from the Windows installation), RFB (RealVNC 5.3.2), and TeamViewer v11. For ICA HDX (Citrix XenDesktop 7.5), we created a local installation on a server with one CPU core at 2.4 GHz and 4 GB RAM.

**Fig 1:**
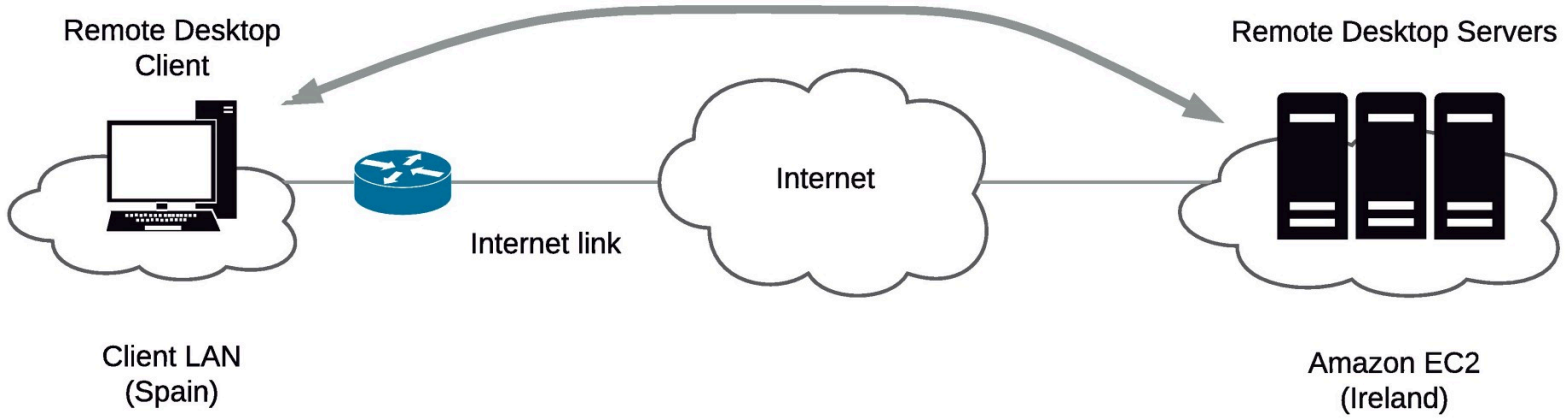
Experimental scenario for evaluation of RD protocols.

All the tests were performed using the same desktop PC as the client in our local network at the Public University of Navarre in Spain. For each remote desktop service, the most recent client version was selected. The Amazon WorkSpaces client v.2.1 was used to control the first virtual server (the one in the Amazon cloud). The client for RDP was the one from the Windows 10 installation, using RDP8 as the maximum common version between the client and server. The VNC client was Real VNC Viewer v5.3, and for the ICA protocol, the client from the Citrix XenDesktop 7.5 installation was selected. The local video resolution was 1280×1024 pixels. The setup is displayed in Fig 1.

The access link to our Internet Service Provider (ISP) was recently updated to full-duplex 10 Gb/s. During the experiments, we conducted network bandwidth and delay tests between our local network and the Amazon datacentre in Ireland, achieving average rates of approximately 60 Mb/s downstream and 90 Mb/s upstream. The average measured round-trip time (RTT) was 53 ms. This is an average delay well below the maximum recommended value for interactive applications (150 ms one-way, from ITU-T G.114 recommendation). Based on these measurements, we did not expect any limitation due to the access link.

### User profiles

To measure the performance of remote desktop services, we defined three user profiles similar to those in [40] [41]: office, web browsing, and video user profiles. They present different degrees of interactivity with the desktop and result in varying frequency changes to the desktop output.

We recorded user actions (keyboard presses and mouse movement events) using the Macro Recorder tool [42]. The actions recorded were replayed for each remote desktop environment and each experiment. In this manner, we guaranteed the same user actions for all experiments, with the same timing. The experimental data captured from the user interactions with real remote desktop services was obtained by the authors of this paper acting as the users.

For the office user profile, we recorded the actions of a LibreOffice Writer user. He launched the text editor, wrote text, changed text styles, added images, and saved the document. All these steps required text selections, interaction with menus, and pop-up windows.

For the “web browsing” profile, a user wrote a URL in a web browser and a rich content webpage was loaded. He scrolled around the page and clicked on hyperlinks. Three different webpages were loaded during the recorded test, including a newspaper landing page, a university homepage, and a web containing online courses. These were considerably different in the amount of multimedia content (videos, flash content, animations, and images) included.

The “video” user profile was based on a user viewing several video files. The same video files were reproduced at low and high resolution (from 144p to 1080p) using YouTube video qualities, including scenarios using full-screen mode. Some RD systems use a channel to transfer the multimedia file from the server to the client, whereas others send an on-the-fly compressed version of the video screen, extracted directly from the framebuffer. We expected large differences in video quality from one RD solution to another, especially for the 1080p resolution. Previous published works have used lower resolution video files [17]; today, desktop users require viewing video presentations or video collaboration where high-resolution videos are streamed.

### Experimental setup and performance metrics

We selected metrics for the evaluation of the network usage and QoE. These were based on the network traffic and video streams at both the server and client. A network usage profile is required for any link dimensioning to determine the minimum available capacity required in the path between a set of clients and servers in a VDC scenario. As the network path was not congested during the conducted experiments, we provide a measurement of maximum bandwidth required. The RTT was considered approximately constant for all the experiments as all the servers were co-located in the same datacentre and there was a common client. We attempted to correlate the user perceived quality with network usage, as these were expected to be tightly coupled. The downstream throughput was the principal component, as the upstream flow contained mainly user input (keyboard and mouse), protocol “keepalives”, and acknowledgements. We used tcpdump [43] at the client for the traffic capture and tcpstat [44] for the network traffic analysis.

The service quality experienced by the user of an RD system depends on the video quality and interactivity. A system can apply high compression techniques and produce a low bandwidth stream. The result is a lower video quality due to the high compression rate and a reduced interactivity due to the increased compression delay. Conversely, a system that applies low compression techniques produces higher video quality and lower delays; however, this is realised at the expense of higher traffic bitrates.

The QoE is measured based on the difference between the video stream directly at the video output in the source desktop server and at the destination RD client. We use the PSNR [19] as an objective metric of the difference between both video sources. A large degree of compression results in a reduced PSNR because of the difference between both streams. Large delays result in temporal desynchronization between the flows, again with the result of a reduced PSNR. The PSNR has been used in previous studies on quality in streamed video [45] [46].

The video streams at the RD server and client are recorded simultaneously using Badicam software [47]. Both video streams are compared using EvalVid [19]. EvalVid is a well-known tool for video evaluation in the research community [45] [48] [49] [50]. This tool offers a PSNR measurement designed for the evaluation of video transmission over a network path with losses. For its operation, we were required to synchronize both video streams to compare them. We did this on a frame level by starting the video comparison from the timestamp when a small rectangle in the screen was modified owing to a mouse click. The small rectangle was the only change in the screen and therefore a small compression delay was expected. This delay and the one-way-delay were eliminated owing to this synchronization.

A reduced PSNR between both video streams could be the consequence of a loss of video quality due to a high compression rate at the server. The majority of remote desktop services use TCP at the transport layer and therefore network losses do not introduce video quality degradation. However, network losses (due to their recovery time), network one-way delay variations, and slower compression result in greater delays between the video at the RD server and the client. The result is a stream desynchronization that also produces reduced PSNR values. The remote desktop systems recover quickly from the desynchronization; however, the PSNR has already been locally impacted. EvalVid offers a relation between the measured values of the PSNR and an estimated MOS [19]. The MOS is the standard QoE metric. Table 2 displays this relation, extracted from [19].

**Table 2:** PSNR to MOS mapping and ITU-R quality and impairment scales [19].

PSNR[dB]	MOS	Quality	Impairment
>37	5	Excellent	Imperceptible
32–37	4	Good	Perceptible, yet not annoying
26–31	3	Fair	Slightly annoying
21–25	2	Poor	Annoying
<20	1	Bad	Extremely annoying

For every combination of RD system and user profile, the above-mentioned metrics were recorded. For each experiment, the procedure was: launch traffic capture at the client, launch desktop video capture at the server and the client, and finally, play the recorded user events (using a macro) for the selected user profile. After an experiment completed, both video streams were collected at the same machine, re-synchronized, and EvalVid was executed, obtaining the PSNR and MOS results. Based on the capture of network traffic, a network traffic profile was obtained.

### Statistical model for remote desktop traffic

For scenarios with cloud deployment of remote desktops, the traffic from this service uses the company’s Internet access link. Sizing the required capacity for the access link and its packet buffers is vital for an adequate QoE. This dimensioning requires characterising the statistical behaviour of the remote desktop traffic.

It has been reported for two decades that, contrary to traditional teletraffic theory, Internet traffic cannot be adequately modelled by processes with independent or short-range dependent random variables. High-resolution traffic measurements in LAN and WAN scenarios [51] [52] [53] have indicated that network traffic exhibits Long Range Dependence (LRD), which is a property of self-similar or fractal random processes. Measurements from applications such as the World Wide Web [54] and Variable Bit Rate Video [55] have indicated that they generate traffic that is consistent with self-similarity.

Self-similarity in a random process can be defined based on the autocorrelation function of the aggregated process. Let *Z*(*t*), *t* ∈ *R* be the continuous process of the number of bytes arriving in time interval [0, *t*). Consider the stationary discrete-time process *X* of the number of bytes per time interval *δ* as:

$$\begin{array}{c} X=\left\{X_{k}\right\}=\left\{Z\left(k\delta \right)-Z\left(\left(k-1\right)\delta \right),k\in N,k\geq 1\right\} \end{array}$$ (1)

The process defined as $X^{\left(n\right)}=\left\{X_{i}^{\left(n\right)}\right\}$ is an aggregated process where:

$$\begin{array}{c} X_{i}^{\left(n\right)}=\frac{1}{n}\sum_{k=n(i-1)+1}^{ni}X_{k},n>1,i\geq 1 \end{array}$$ (2)

Let *ρ*^(*n*)^(*j*) with *j* > 1 be the autocorrelation function of *X*^(*n*)^. The process *X* is asymptotically second-order self-similar if the following limit for its autocorrelation function is true:

$$\begin{array}{c} lim_{n\to \infty }\rho^{\left(n\right)}\left(j\right)=\frac{1}{2}\left({\left(j+1\right)}^{2H}-2j^{2H}+{\left(j-1\right)}^{2H}\right) \end{array}$$ (3)

where *H* is the Hust parameter. For 1/2 < *H* < 1 the autocorrelation function decays slowly, being not summable, and we say that *X* presents long-range dependence.

This is a definition of self-similarity as an asymptotic property (it only occurs when *n* → ∞). There is a timescale (*δ*) beyond which the traffic behaves as a stationary Gaussian self-similar process with constant *H* parameter, whereas at short scales it is better described with complex multi-fractal models [56] [57]. For large traffic aggregation levels, parsimonious modeling based on fractals suck as Fractional Brownian Motion (FBM) are predominant [58] [59] [60].

A FBM *F*_*H*_(*t*) is a Gaussian process that satisfies:

$$\begin{array}{c} E\left[F_{H}\left(s\right)F_{H}\left(t\right)\right]=\frac{1}{2}{\left(|t\right|}^{2H}+{\left|s\right|}^{2H}{-|t-s|}^{2H}) \end{array}$$ (4)

An Fractional Gaussian Noise (FGN) is defined as the increments of an FBM. An FBM is used as a model for the cumulative arrival process *Z*(*t*) where the FGN models the arrival process *X* of bytes per interval.

For a process with independent increments whose marginal distribution has variance *σ*^2^, the aggregated process with level *δ* presents a variance *δσ*^2^. However, for an FBM the variance in the aggregated process $\sigma_{\delta }^{2}$ is [61]:

$$\begin{array}{c} \sigma_{\delta }^{2}=\delta^{2H}\sigma^{2} \end{array}$$ (5)

For *H* > 0.5 the result is larger variability in the arrival process of traffic to a network link, longer delays waiting in queue and larger packet loss probabilities [58].

In this paper, we study the long-range dependence in remote desktop traffic based on estimations of the Hurst parameter. We evaluate its value for different protocols and user profiles and its influence for large user aggregation levels.

In the following sections, we first evaluate Amazon WorkSpaces in terms of transfer rate and QoE. Then, this is compared with the other remote desktop protocols.

## Evaluation of PCoIP (Amazon WorkSpaces)

This section presents the evaluation results for the PCoIP protocol as deployed in Amazon WorkSpaces DaaS. It is a novel scenario, offering a massive deployment for the provision of virtualized desktops in the cloud. We identify the network and server requirements for each user profile as defined in a previous section and evaluate the resulting QoE. We model the user traffic as a self-similar arrival process, with different parameters for each user profile, which influence network link dimensioning. In a later section, we compare the results from PCoIP (Amazon WorkSpaces) to RDP, VNC, ICA, and TeamViewer.

### Transfer rate

The access-link available bandwidth and link usage are fundamental characteristics as they limit the number of users for which remote desktop services can be deployed. Peak bitrate and its average are strongly dependent on user behaviour.

**Fig 2:**
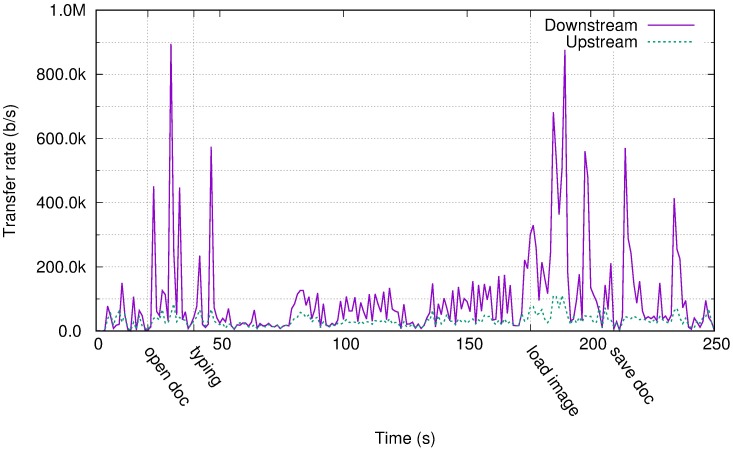
Transfer rate for the office profile in Amazon WorkSpaces.

Fig 2 displays the time series of link bandwidth usage for an experiment with a user with an office profile. Principal events are marked in the time axis. As detailed in section 3 the user performs several tasks, opening and editing a document. The user performed several tasks while opening and editing a document. The main events are marked in the time axis. As expected, the upstream requirements are low compared to the downstream requirements. For 99% of the time, the upstream link rate usage was less than 100 Kb/s, whereas the downstream link rate approached 900 Kb/s when large changes occurred on the screen, for example, when a new document window was opened or a large image was inserted.

Traffic behaviour was consistent with Amazon recommendations for Amazon WorkSpaces [62], where it states that “*the network connection should provide at least 300 Kb/s, with capability to provide over 1 Mb/s when viewing video or using graphic-intensive applications*”. We must note that text editing is typically not a graphic-intensive application; however, it presents spikes in network usage consistent with this recommendation. It is demonstrated later that for video playback, the network requirements are considerably greater than those recommended.

**Fig 3:**
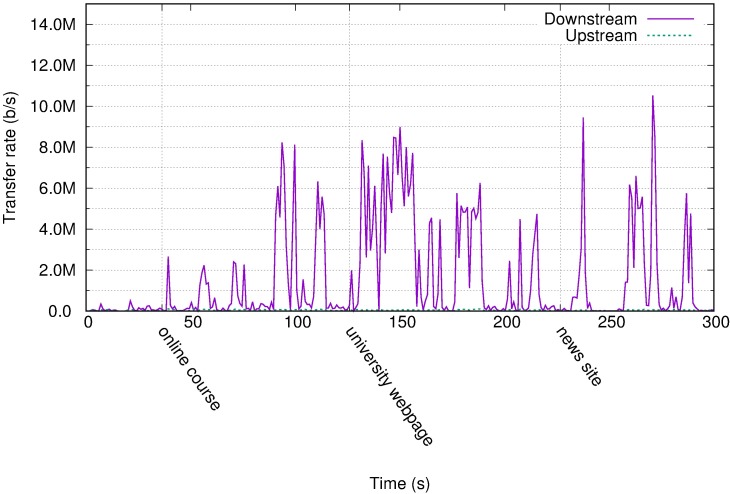
Transfer rate for the web profile in Amazon WorkSpaces.

Fig 3 displays the traffic profile for a case of a web browsing user. The principal events are marked in the time axis. The first site visited was a web page containing online courses. The user logged in, located a course, and viewed a PDF document in the browser window. The actions of receiving the screen containing the PDF and scrolling through this screen are clearly marked with spikes reaching 8 Mb/s.

The second visited web site was a university landing page. The user browsed through the news and information regarding the academic degrees offered. There were moving banners and automatic slideshows in the web page that resulted in continuous screen changes and therefore sustained traffic rates greater than 6 Mb/s. This is a clear indication of how rich content in a web page based on JavaScript, harmless in a local desktop environment, can result in high bandwidth requirements in a remote desktop deployment. The changing images on the screen were not large files, yet because of the animations, they became a video stream.

The third visited web page was the landing page of a news site. The user scrolled the news headers and visited some of these. The page did not contain moving banners and hence did not result in sustained high bitrates. However, the multimedia linked files (images) were large, surpassing more than 9 Mb/s for some of the screen updates. The main insight from Fig 3 is how apparently low profile web pages can become traffic intensive in a RD deployment owing to remotely rendered animations.

**Fig 4:**
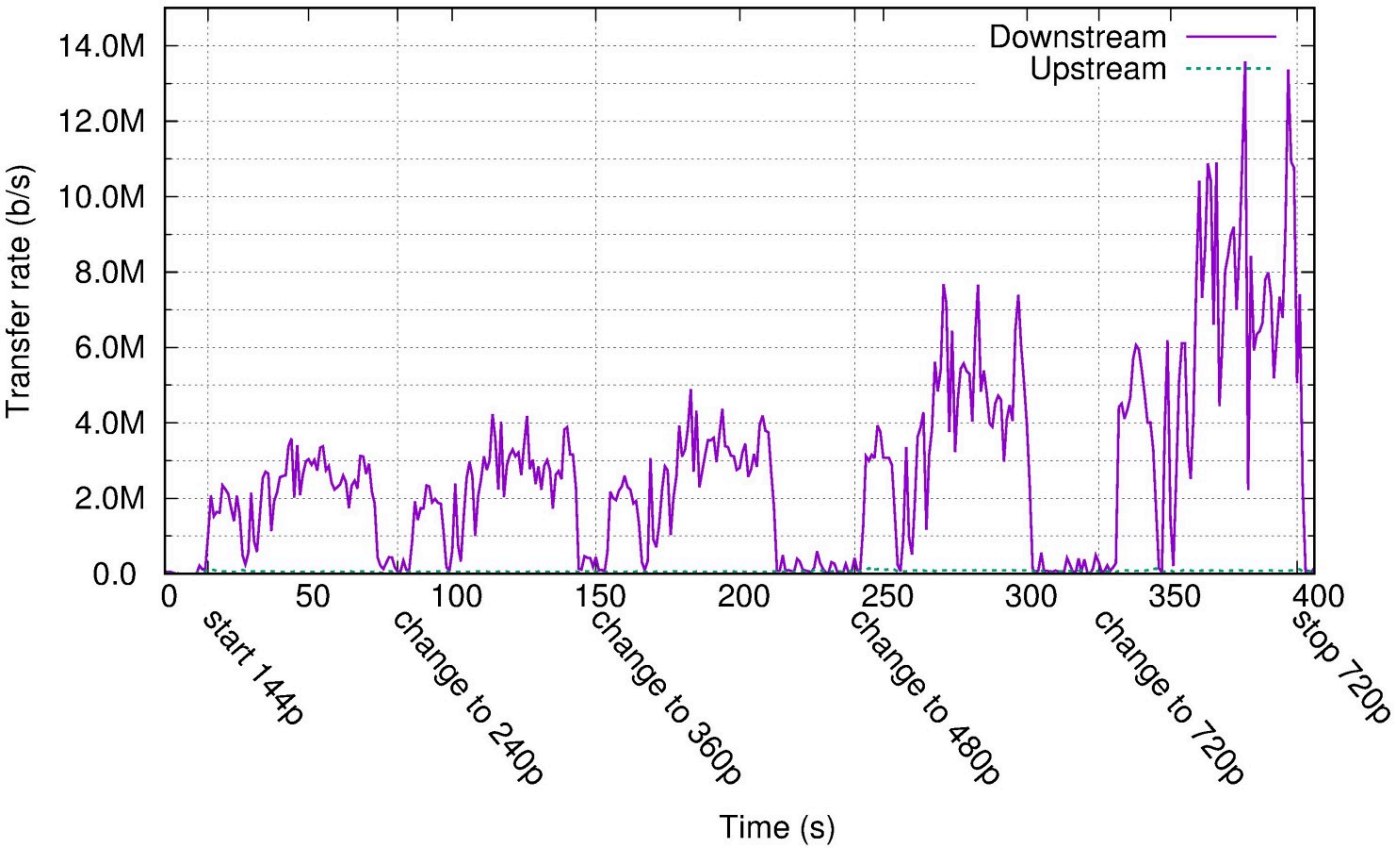
Transfer rate for the video profile in Amazon WorkSpaces with different video sizes.

Fig 4 displays the traffic rate for one experiment with the video user profile. The same video file was viewed at different resolutions from the YouTube webpage. The x-axis in Fig 4 displays the approximate time when the user changed the resolution. PCoIP did not transfer the video file for local playback at the client. Starting from a 144p video resolution using more than 2 Mb/s, the link usage reached 10 Mb/s for the 720p version owing to the larger screen surface experiencing quick changes. These results are in contrast to the Amazon recommendations of between 300 Kb/s and 1 Mb/s for video intensive applications [62].

**Fig 5:**
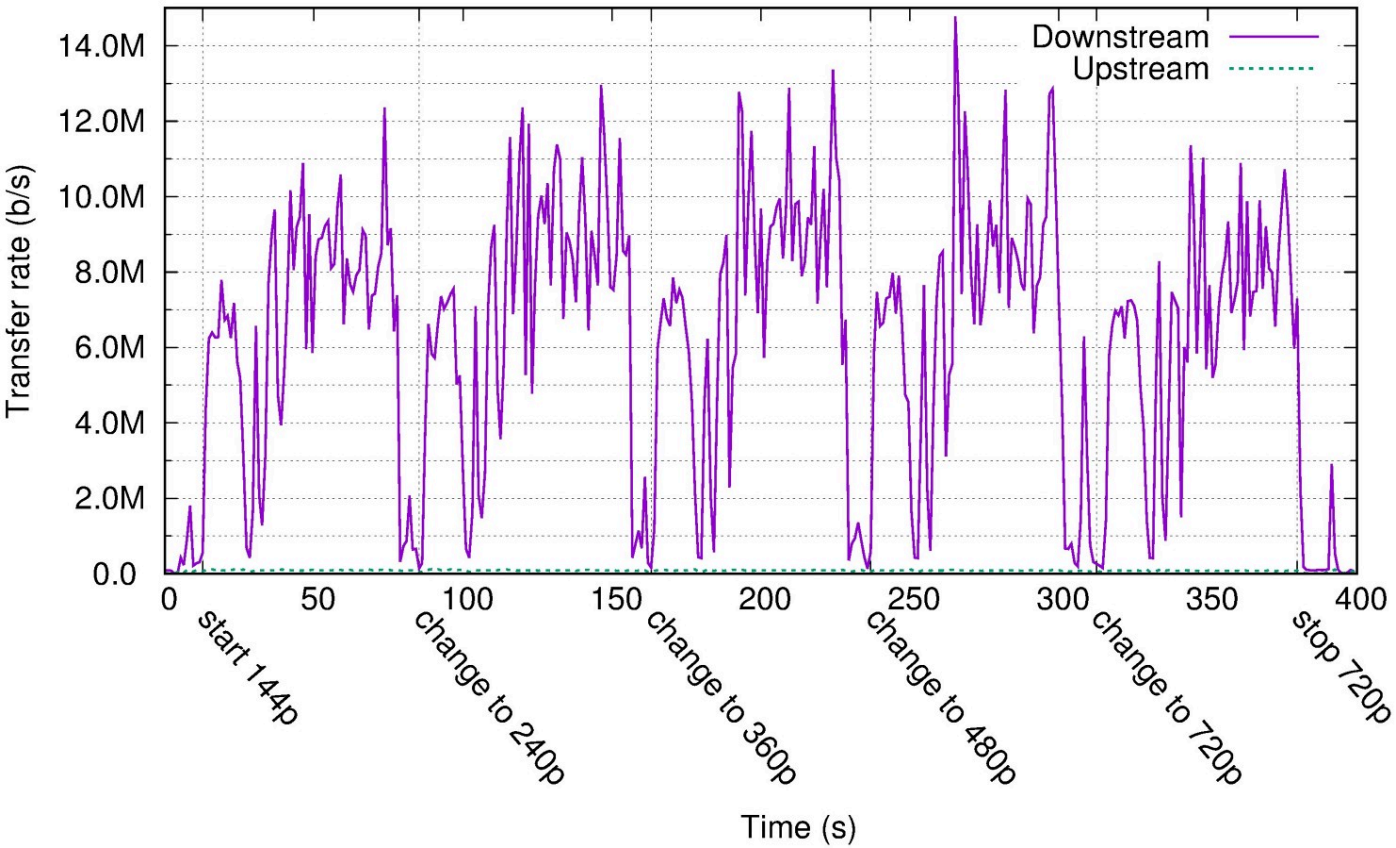
Transfer rate for video profile in Amazon WorkSpaces with full screen.

If the user presents the video in full screen mode, the transfer rate is similar for every video file resolution. The video playback program uses interpolation techniques to produce a higher resolution video stream that fills the screen, instead of presenting a simple scaled version of the video. Therefore, changes occur everywhere in the screen and, as indicated in Fig 5, the compressed flow to the client presents a similar transfer rate, independent of the original video resolution.

**Fig 6:**
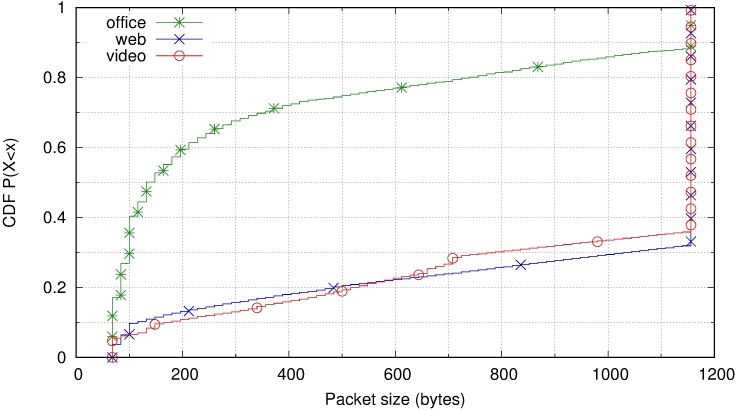
Packet size cumulative distribution function for three user profiles in Amazon WorkSpaces.

A parameter related to the transfer rates is the packet size. Fig 6 displays the cumulative distribution function of packet sizes for the three user profiles in the Amazon WorkSpaces scenario. For the web browsing and video profiles, 70% of the packets had the maximum size, that being 1156 bytes, considerably less than the Ethernet Maximum Transmission Unit (MTU) of 1500 bytes. This maximum size avoids fragmentation of packets passing through VPNs or tunnels. It is preferable to avoid fragmentation as fragmentation results in a higher impact of the losses on performance. Because web browsing and video profiles have higher transfer rates, maximum-sized packets are used. In the office profile, the packet sizes were more variable, with a greater percentage of small packets because the information sent corresponded to refreshments of smaller screen zones.

In conclusion, the transfer rates identified for Amazon WorkSpaces differ substantially from the rates that Amazon itself recommends for the deployment of its services [62], with a transfer rate approximately 300–1000 Kb/s. This recommendation is valid for an office profile; however, for the web browsing and multimedia profiles it is clearly insufficient because these profiles frequently approached rates of 5–10 Mb/s. Even in the case of visualisation of low-resolution small videos, 1 Mb/s would not be sufficient; it would require 2–3 Mb/s. A short available bandwidth could automatically mean a loss of interactivity in the service (it is not possible to send the screen in real time) and a loss of quality of image (using stronger compression schemes with losses).

### Quality of experience

We compared the desktop video stream recorded at the server (sent) and the client (received). Highly lossy compression and delay variations result in changes between both video streams. We obtained a PSNR time series of these changes using EvalVid. From this PSNR, a corresponding MOS value was obtained from Table 2.

**Fig 7:**
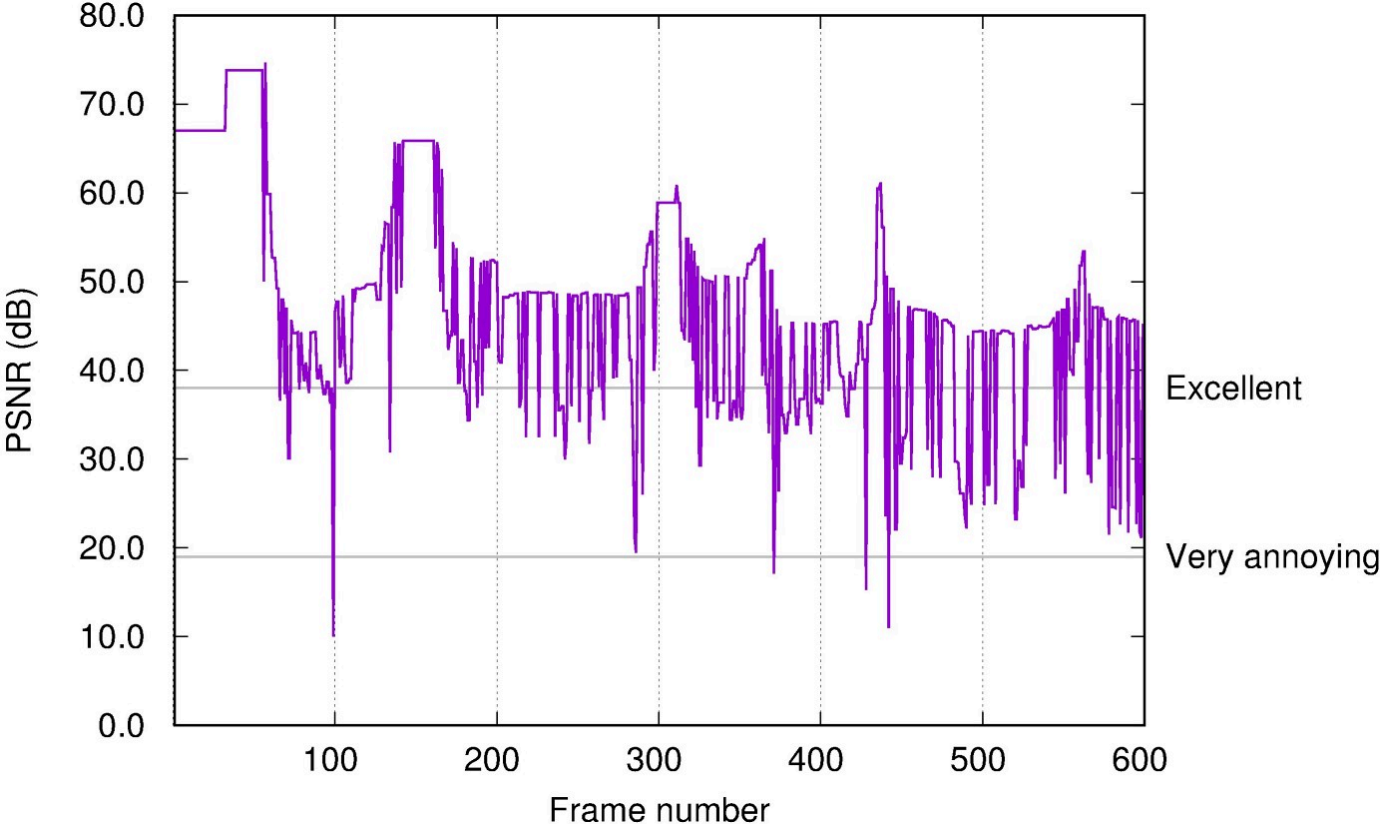
PSNR time series for video profile in Amazon WorkSpaces.

Fig 7 displays an example of 600 frames (20 s) of PSNR time series for the video user profile while the user was viewing a 480p video file. The minimum PSNR values are due to transitions between scenes where large changes in the screen occur frequently. In these situations, the amount of data to be sent is greater and hence it arrives at the client with a greater delay. Even without loss of video quality, there is a higher delay (worse perceived quality) that is measured by the desynchronization between both streams and hence, a lower PSNR; Frame 99 in Fig 7 presents the lowest PSNR value. This is a result of the scene transition. Similar situations occur for other user profiles when large changes in the screen are required (i.e., a large window appears, the user performs a rapid scroll, or a new web page is rendered).

However, in this scenario without bandwidth limitations, image quality is, in general, “Good” or “Excellent”. Even though there are differences between the source and destination streams, there are no noticeable compression artefacts. The differences could be noticeable through a heatmap, however not directly by the eye of a user.

**Fig 8:**
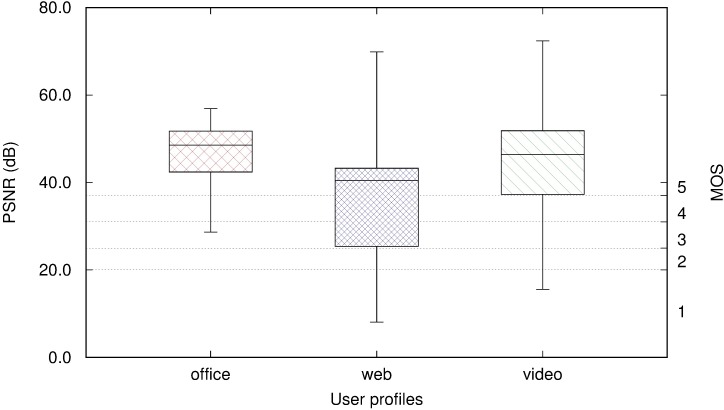
Boxplot of PSNR for three user profiles in Amazon WorkSpaces.

We summarise the PSNR time series for each user profile using the first, second (the median), and third quartiles. We display these values in Fig 8, with the maximum and minimum values of PSNR in a boxplot [63] and their corresponding MOS values in the right vertical axis. The office user profile (leftmost boxplot in Fig 8) obtained a first quartile greater than 40 dB, which means that more than 75% of the time the quality was considerably greater than MOS 5 (“Excellent” quality). The video user profile (rightmost boxplot in Fig 8) presents a higher variability, however it maintains “Excellent”quality for 75% of the time. Finally, the web browsing profile achieved the poorest MOS, less than “3” (“Fair”) for more than 25% of the time.

Surprisingly, the PSNR values were less in the web browsing profile compared to the video profile. Moreover, the web browsing profile resulted in a higher data rate than the video profile. The web browsing profile using images, animations, and advertisements was more demanding in PCoIP DaaS than video streaming.

### Long-range dependence in PCoIP traffic

Applications such as the web or variable bit rate video generate self-similar traffic. Therefore, it was expected that remote desktop traffic would exhibit this property. We evaluated the presence of this property by estimating the Hurst parameter for the traffic arrival process. Many of the proposed algorithms for the Hurst parameter estimation are based on the variance aggregation plot, R/S (rescaled range) statistic [54], periodogram or decomposition of the random process based on the wavelet transform [64], among others. In this paper, we use the variance aggregation plot, similar to many previous works [54] [58].

**Fig 9:**
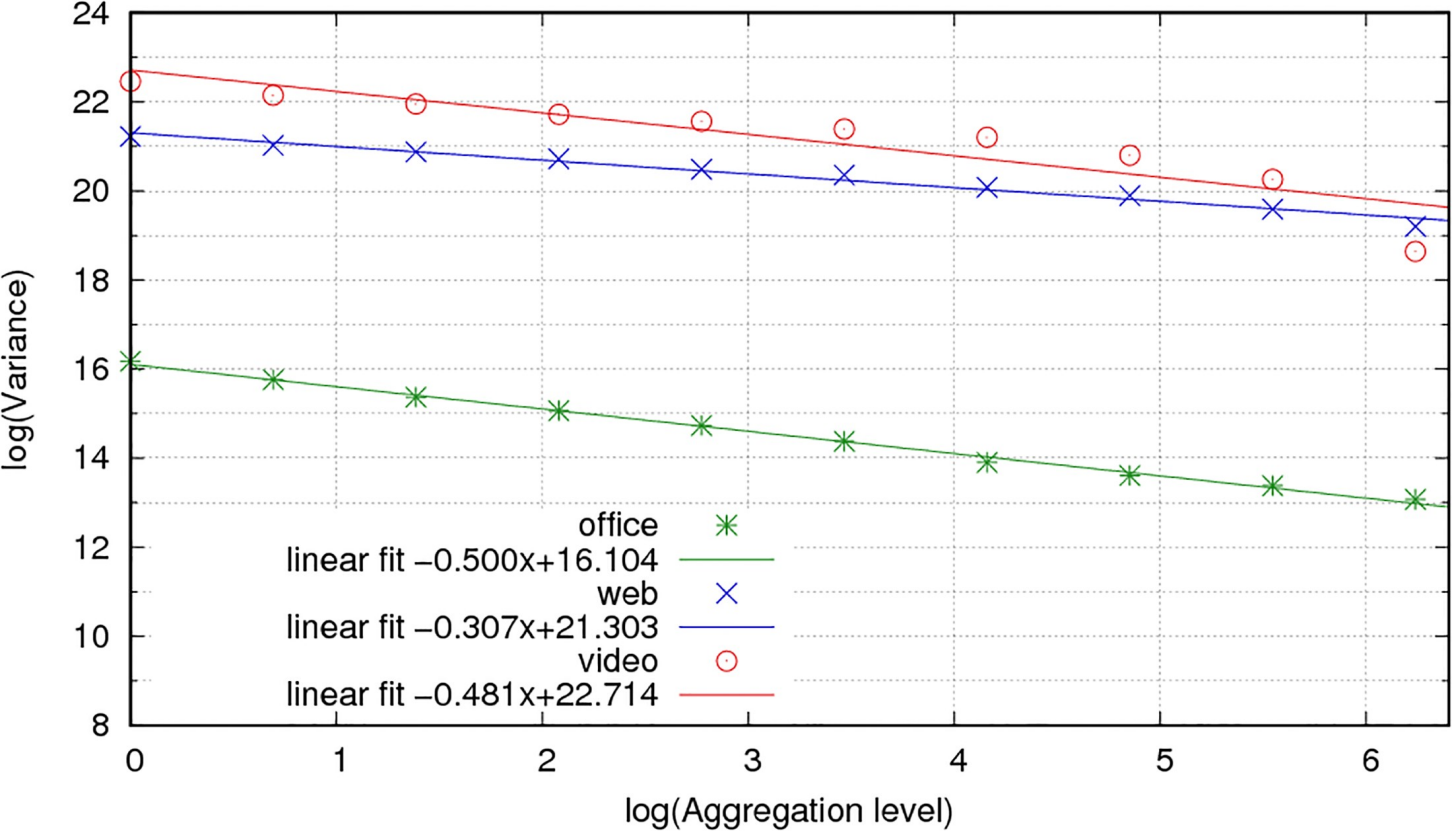
Variance aggregation plot for PCoIP traffic.

Fig 9 displays the variance aggregation plots for PCoIP traffic and the three different user profiles. In a pure (non-asymptotic) self-similar process, the plot in a logarithmic scale is a straight line. The Hurst parameter is therefore estimated from the slope of this line. We use least squares regression to compute the slope *α* for each data set. The resulting Hurst parameter is computed as *H* = (1 + *α*/2). Table 3 presents the estimated values of *H* and the coefficient of determination in the regression (*r*^2^), measuring the quality of the fit.

**Table 3:** Estimation of Hurst parameter for PCoIP traffic.

User profile	Estimated H	*r*^2^
Office	0.750	0.996
Video	0.760	0.829
Web browsing	0.846	0.981

The linear fit is acceptable for the office and web users, which indicate clear long-range dependence (*H* > 0.5). For the video user, the scaling changes and is not as well fit by a strictly self-similar process (FBM). It continues to provide an estimation of H greater than 0.5 for the scales of interest and indicates that the model is sufficiently accurate.

We can model PCoIP traffic for the office and video profiles using an FBM process with the Hurst parameter close to 0.75, and with parameter 0.85 for the web browsing profile. In comparison to a process with independent increments, a self-similar process presents a lower decay of the variance in its marginal distribution with the aggregation level. From [65], we also know that the queue length in a network link that receives a packet arrival process modelled by an FBM strongly depends on H. Let L be the queue length, then the probability of queue occupancy presents an asymptotic lower bound:

$$\begin{array}{c} P\left(L>x\right)\approx e^{-cx^{2-2H}} \end{array}$$ (6)

where c is defined in Eq 7, *ρ* is the link utilisation factor, and *m* is the mean input traffic.

$$\begin{array}{c} c=\frac{m^{2H-1}{\left(\frac{1-\rho }{\rho }\right)}^{2H}}{2a}{\left[{\left(\frac{1-H}{H}\right)}^{H}+{\left(\frac{H}{1-H}\right)}^{1-H}\right]}^{2} \end{array}$$ (7)

Compared to a traffic arrival process with short-range dependence, a self-similar arrival process modelling the remote desktop traffic results in a slower decay in the tail of the survival function of the queueing delay in the routers (Eq 6). Larger buffers or higher speed links are required to obtain similar results of losses and delay and therefore provide a similar quality owing to network transport.

## Comparison of performance metrics for remote desktop protocols

In this section, we compare the PCoIP protocol and its implementation in Amazon WorkSpaces to RDP, TeamViewer, VNC (RFB), and Citrix ICA protocols. We follow the same procedure used in the previous section and present the results for network bandwidth usage, self-similarity, and QoE for each of the three user profiles.

### Transfer rate

**Fig 10:**
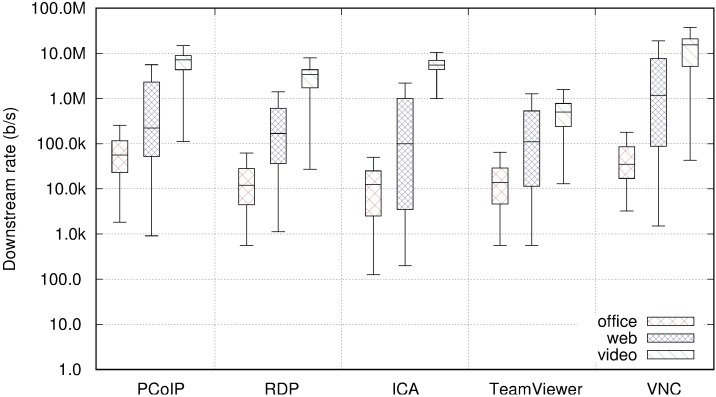
Comparison of downstream rate per user profile and remote desktop protocol.

Fig 10 displays the downstream rate for each remote desktop system and each user profile. We have used boxplots representing the minimum, maximum, and quartiles for the bitrate obtained from each experiment. The leftmost plots in Fig 10 correspond to the results presented in Figs 2, 3 and 4 for the PCoIP case.

The results are consistent among the five remote desktop systems. The user profiles with larger and more frequent screen changes require more link capacity (web and video profiles); however, the rates vary substantially among the different systems. Attention must be addressed to the logarithmic scale employed for the downstream rate in Fig 10, as small steps in the figure represent large changes in link capacity requirements. For example, the median traffic rate for the web browsing profile using TeamViewer is 100 Kb/s whereas using VNC it is close to 700 Kb/s. VNC and PCoIP present the highest bitrates. These RD systems transfer bitmaps from the server to the client. In comparison, RDP and ICA transfer system graphics commands, which result in lower bandwidth requirements when direct video playback is not involved. In these cases, when video is viewed, RDP and ICA can transfer the file for local playback at the client. TeamViewer achieves one of the lowest rates, especially for the video user profile, however, as will be demonstrated later, this is a consequence of higher video compression, including loss of video quality and reduced QoE

Table 4 displays the average transfer rates for the upstream direction. The rates are low compared to the downstream rates, as was the case for PCoIP. VNC should be observed, attaining an average upstream rate of 320 Kb/s in the video profile, which must be compared to a median of 20 Mb/s downstream. A 20 Mb/s TCP flow in one direction requires a considerable amount of traffic in the opposite direction for TCP acknowledgements, hence this upstream rate is not due to application level traffic in the upstream direction; rather, it is due to transport layer control traffic.

**Table 4:** Average upstream rate per remote desktop protocol.

Protocol\Profile	Office profile	Web profile	Video profile
PCoIP	32.18 Kb/s	45.48 Kb/s	83.46 Kb/s
RDP	14.10 Kb/s	32.74 Kb/s	149.01 Kb/s
ICA	13.51 Kb/s	40.12 Kb/s	171.63 Kb/s
TeamViewer	12.05 Kb/s	10.90 Kb/s	14.12 Kb/s
VNC	14.56 Kb/s	111.26 Kb/s	320.85 Kb/s

**Fig 11:**
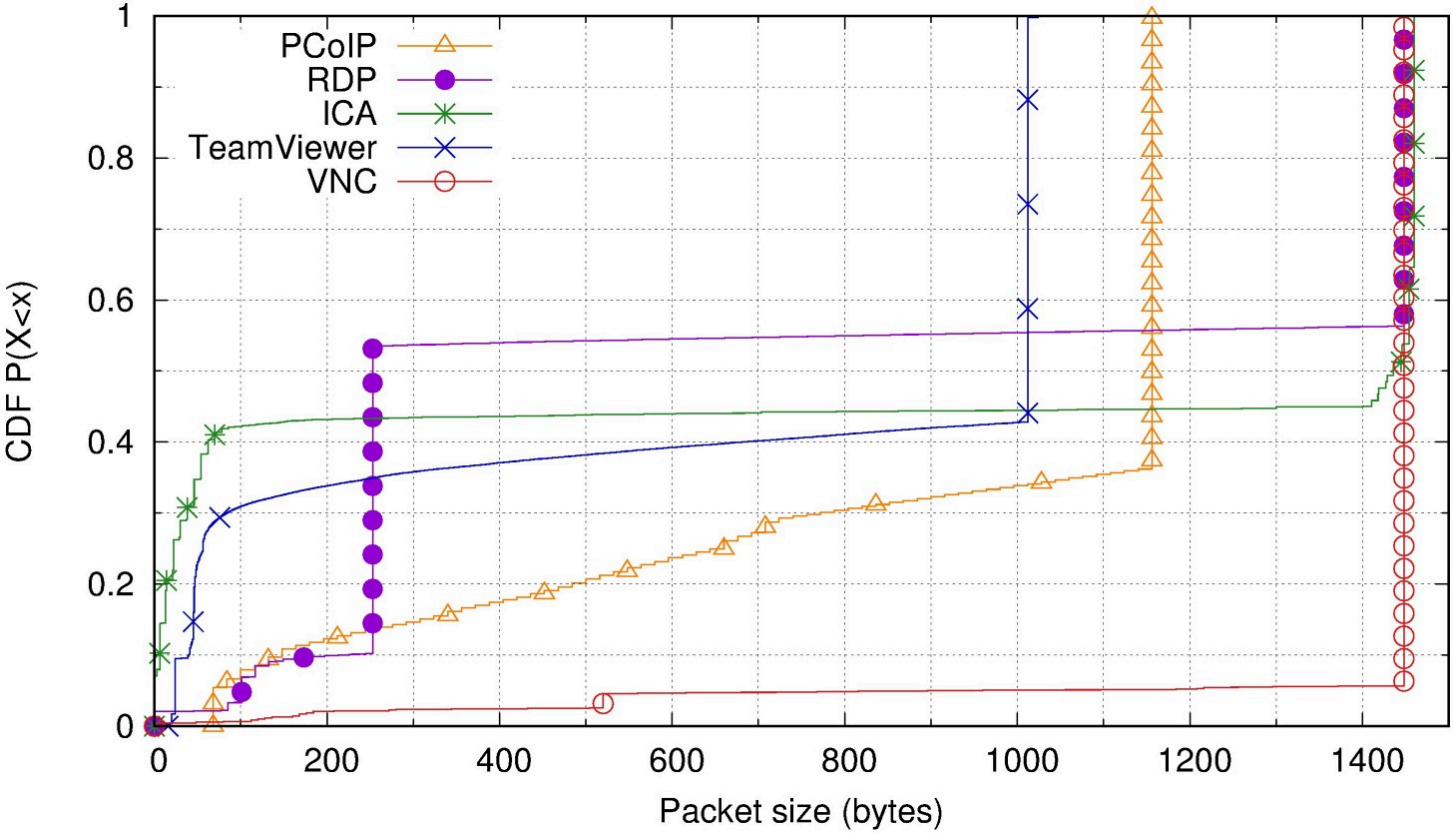
Packet size cumulative distribution function for all remote desktop protocols.

Regarding packet sizes, Fig 11 displays the cumulative distribution function of downstream packet sizes for all user profiles and each remote desktop protocol. The most notable aspect is that the remote desktop protocols that use UDP as a transport protocol (PCoIP and TeamViewer) do not reach the maximum packet size that the path MTU allows. This could be related to an interest in avoiding fragmentation in the event of traffic that must traverse VPNs or tunnels between the client and server. For systems that use the TCP transport protocol (RDP, ICA, and VNC), the application has no control over how data is packetized. TCP sends packets of the maximum size allowed by the path MTU. Note also that VNC has a higher percentage of large packets, which is consistent with the fact that it consumes more bandwidth than the others.

### Quality of experience

**Fig 12:**
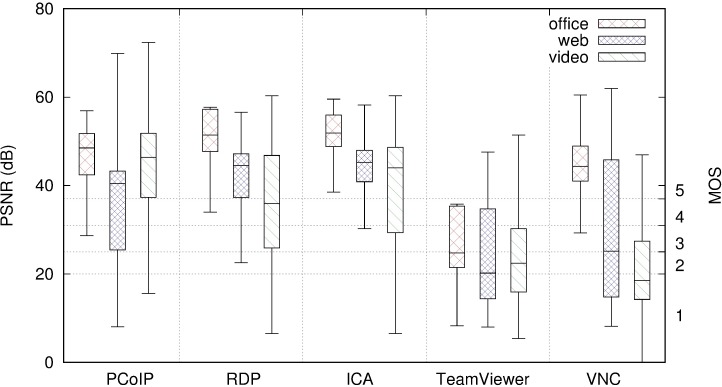
Comparison of PSNR per user profile and remote desktop protocol.

Fig 12 displays the PSNR and corresponding MOS value for each RD system and the three user profiles. The leftmost part of the figure corresponds to the values presented in Fig 8 for the PCoIP scenario. Values of PSNR above 37 dB correspond to the maximum MOS value of “5” (“Excellent” quality).

TeamViewer consistently provided lower values of MOS than the other RD systems. This is because of the lossy compression it applies. For the office user profile, the other RD systems analysed offered excellent quality (MOS “5”) for at least 75% of the frames, whereas TeamViewer never achieved this quality and its median value was in MOS “3” (“Fair” or “Slightly annoying”).

The web browsing user profile demonstrated the highest variability in quality for those protocols that send bitmaps from the server to the client (VNC, PCoIP, and TeamViewer). TeamViewer and VNC provided median MOS values of “2” and “3” for this profile, whereas PCoIP, RDP, and ICA remained above MOS “5” at least 50% of the time.

For the video user profile, fast screen changes have an important influence on QoE because of the additional delay they introduce. VNC and TeamViewer offered the lowest qualities whereas PCoIP maintained an MOS greater than “4” more than 75% of the time, even though they all employ bitmap transfers.

TeamViewer demonstrated a reduced MOS because it increases the compression degree when there are rapid changes in the image. It prioritises a fast screen update at the client, at the cost of a lower image quality. The comparison of the video feed at the server and the client in these situations results in a reduced PSNR and hence, a lower MOS value.

VNC not only suffers delays due to a greater amount of data to transfer on fast screen changes but also renders the screen as it receives the data for different sections. The result is that a part of the screen could be displaying a previous video frame and the remainder displaying the new frame. The resulting PSNR of comparing the video feed at the server and the client is seriously hampered in these situations, providing a reduced MOS value.

### Self-similarity and link provisioning

**Fig 13:**
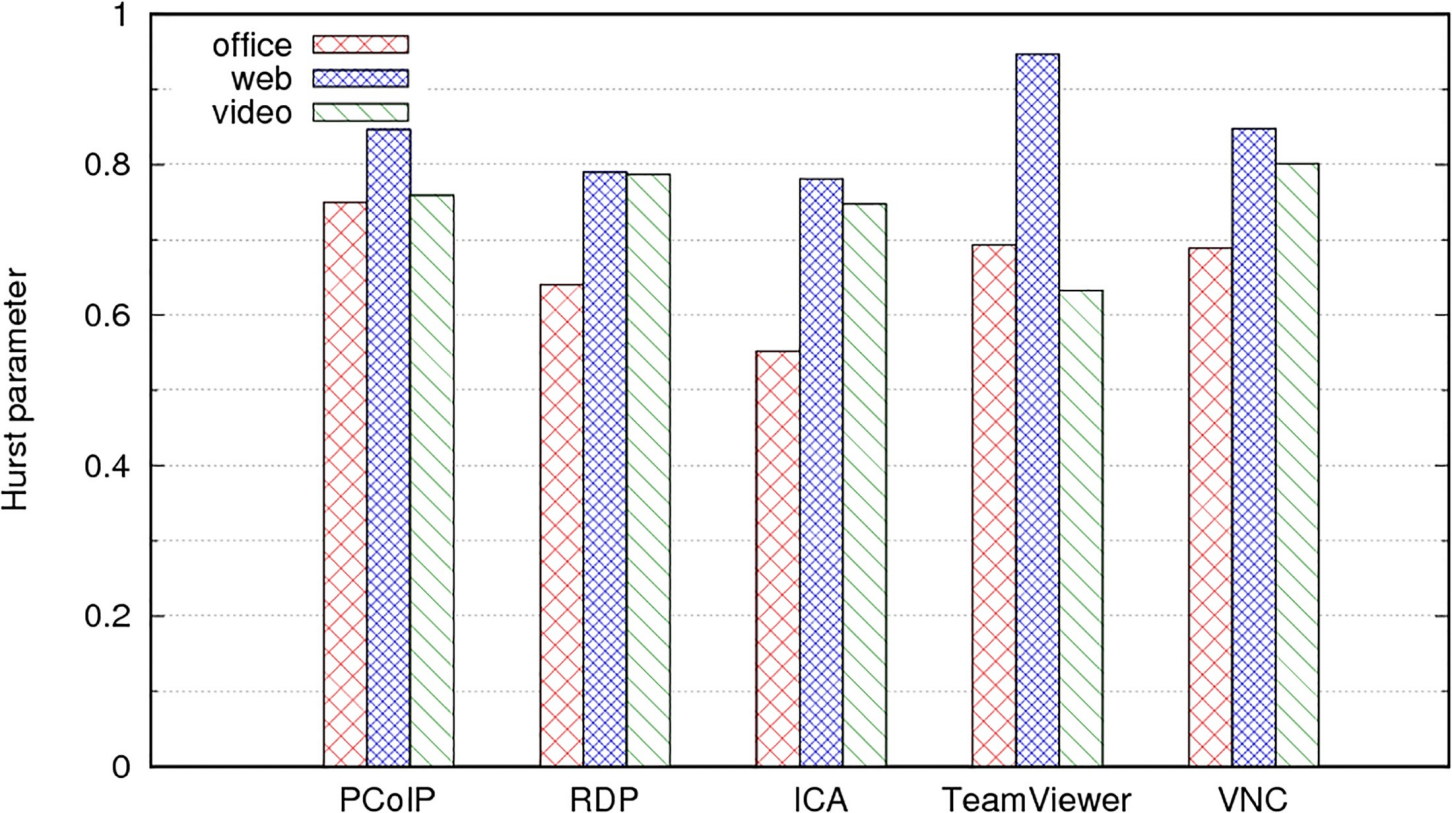
Values of H for different remote desktop protocols and user profiles.

Table 5 and Fig 13 display the Hurst parameter for the different remote desktop protocols and user profiles (apart from PCoIP, which was presented in Table 3). In Table 5, they are sorted by user profile; Fig 13 presents them grouped by protocol.

**Table 5:** Estimation of Hurst parameter for different remote desktop protocols and user profiles.

Protocol	User profile	H	*r*^2^
RDP	Office	0.640	0.992
ICA	Office	0.553	0.992
TeamViewer	Office	0.693	0.918
VNC	Office	0.689	0.975
RDP	Web browsing	0.790	0.976
ICA	Web browsing	0.781	0.972
TeamViewer	Web browsing	0.946	0.870
VNC	Web browsing	0.848	0.981
RDP	Video	0.787	0.971
ICA	Video	0.748	0.971
TeamViewer	Video	0.733	0.979
VNC	Video	0.801	0.929

The office profile creates the traffic process with an H value closest to 0.5 or closest to independent increments, except for PCoIP traffic (Table 3, *H* = 0.75). Conversely, the web user profile creates the traffic with the greatest value of H or the strongest long-range dependence. This consistent behaviour implies that the reason for the LRD is not related as much to the characteristics of the remote desktop protocol as it is to the user actions. For any of these protocols, the web users are those who create the traffic with the strongest LRD and therefore, the poorest behaviour in router queues. Although the video users present the highest average bit rates (Fig 10), their traffic is less bursty than the remote desktop traffic for the web users, therefore link buffers require less over-provisioning for video users.

These results apply to the traffic from a single remote desktop user. In a scenario where all the employees in a company are using remote desktop services, the Internet link must support the multiplex of traffic from all these users. The amount of link capacity or the size of packet buffers in the access router must be determined based on the aggregated traffic.

For a network link that aggregates the traffic from a large population of remote desktop users, we can estimate the Hurst parameter for the aggregated traffic from the FBM model for each user traffic process. We consider two different cases to evaluate the self-similarity in the aggregated traffic. In the first scenario, the remote desktop users are modelled with the same user profile (all are considered office users, video users, or web users). In the second scenario, we consider a mixture of users from the three different profiles.

We computed the average traffic, variance, and Hurst parameter for every combination of protocol and user profile. From these parameters, we can generate synthetic FBM traffic traces using one of the existing FBM generation techniques. For this paper, we used the Random Midpoint Displacement (RMD) method, a fast and efficient generation method adequate for qualitative studies [66]. For every combination of remote desktop protocol and user profile, we created 90 FBM traces. We multiplexed all the traces from the same protocol scenario and user profile. The resulting traffic models the situation where a medium-sized company with 90 users simultaneously use cloud remote desktop services where all users are from the same profile.

**Fig 14:**
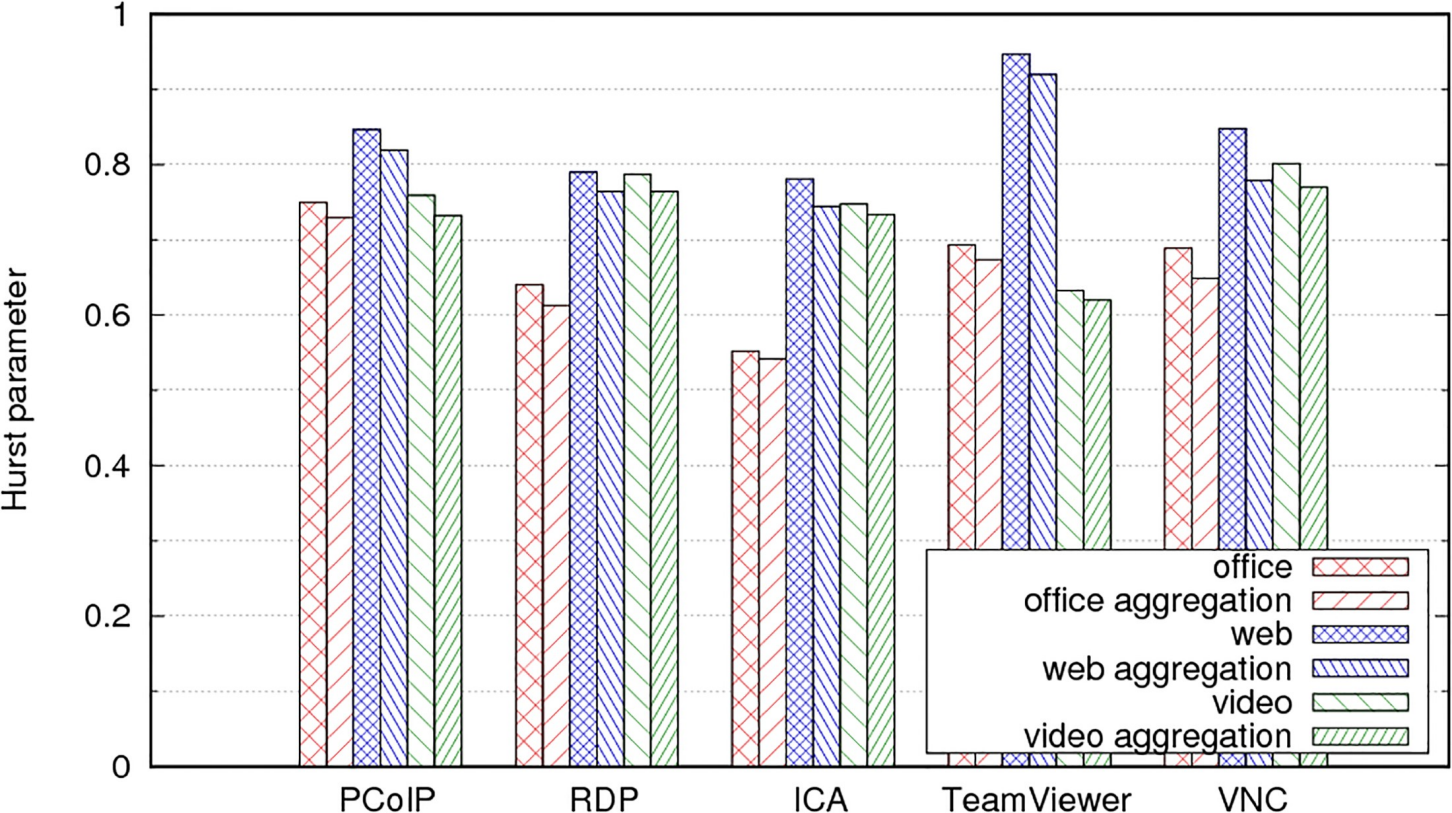
Values of H comparing one user and multiplex of 90 users.

Table 6 displays the estimated Hurst value (using the variance aggregation plot method) for each scenario. As expected, if all the users are from the same profile, the resulting processes tend to the same value of H [67] [68]. Fig 14 compares the value of H for a single user and aggregation of 90 independent users from the same profile and protocol. The reduction in H is minimal for every scenario. Of course, there is also a reduction in variance owing to the aggregation process; however, as indicated in Eq 5, the reduction is less, the higher the value of H.

**Table 6:** Estimation of Hurst parameter for aggregation of 90 users.

Protocol	User profile	H	*r*^2^
PCoIP	Office	0.730	0.9996
PCoIP	Web browsing	0.819	0.9998
PCoIP	Video	0.732	0.9999
RDP	Office	0.613	0.9997
RDP	Web browsing	0.764	0.9997
RDP	Video	0.764	0.9995
ICA	Office	0.542	0.9998
ICA	Web browsing	0.745	0.9977
ICA	Video	0.733	0.9987
TeamViewer	Office	0.674	0.9998
TeamViewer	Web browsing	0.920	0.9995
TeamViewer	Video	0.620	0.9993
VNC	Office	0.649	0.9999
VNC	Web browsing	0.779	0.9925
VNC	Video	0.770	0.9995

In the case of a mixture of processes with different values of H (different user profiles), it has been demonstrated that the resulting process is dominated by the largest value of H in the mix [58]. However, as this is an asymptotic property and each user profile presents different bit rates and variabilities, it is not a simple task to predict the expected reduction in long-range dependence depending on the mixture and number of users.

To compare to the previous homogeneous case, we again multiplexed 90 users for each protocol; 30 users from each of the users profiles. This means that, for example, we created 30 FBM traffic traces using the parameters from ICA office users and multiplexed them to 30 FBM traffic traces from ICA video users and 30 FBM traces from ICA web users. Table 7 displays the estimated value of H from the resulting traffic trace for each protocol.

**Table 7:** Estimation of Hurst parameter for mixture of 30 users from each profile.

Protocol	H	*r*^2^
PCoIP	0.755	0.9992
RDP	0.759	0.9992
ICA	0.642	0.9981
TeamViewer	0.836	0.9817
VNC	0.785	0.9997

**Fig 15:**
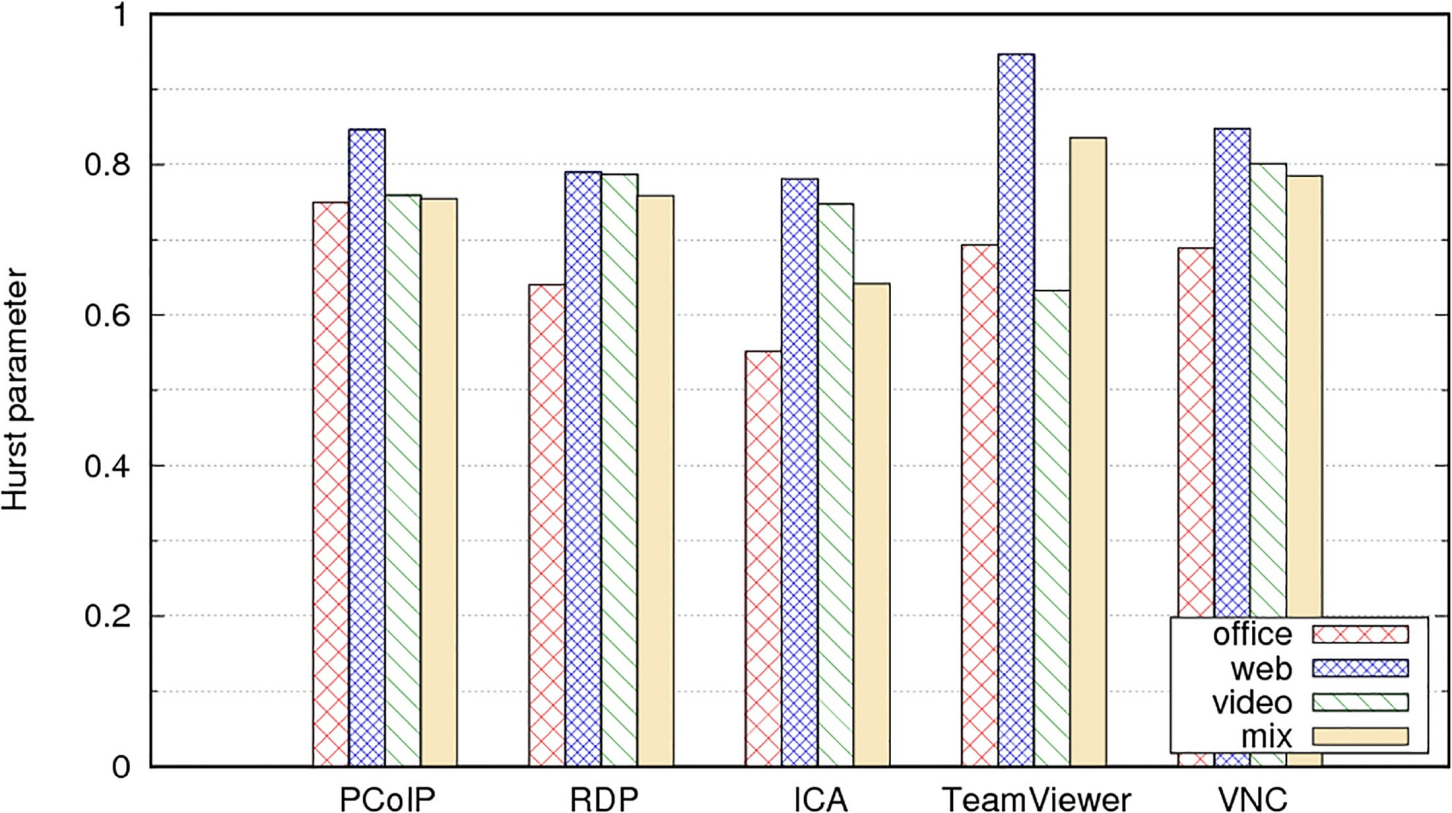
Comparison of values of Hurst parameter for each user profile and mixture of 30 users from each one.

The values of H in the multiplex are not always near the largest H in the mixed set; however, they are always in the range of values in the mixture (see Fig 15). For example, for the ICA protocol, the values for the office, video, and web profiles are 0.542, 0.733 and 0.745, respectively; the traffic mix presents H approximately equal to 0.642, which is not as high as the value of 0.745 for the web profile. For VNC traffic, the values of H in the mix are 0.649, 0.77 and 0.779; the resulting traffic process presents H approximately equal to 0.785, which is similar to the largest value in the mix.

### Quality of experience vs. traffic characteristics

The final evaluation considers the opposing metrics of bandwidth usage and QoE. Typically, a higher quality requires greater bitrates; hence, the tradeoff of achieving the best quality with the lowest bitrate is important.

**Fig 16:**
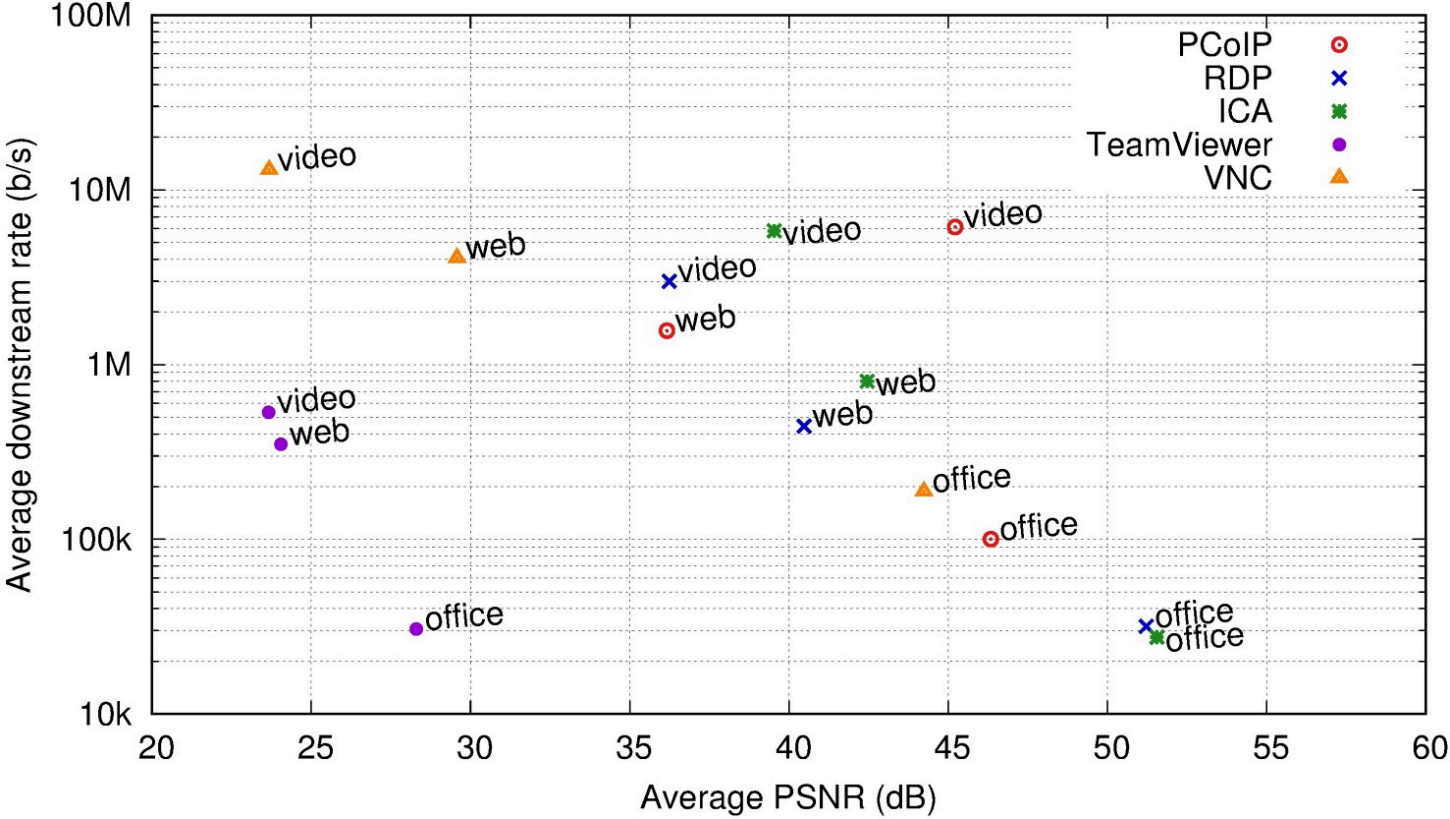
Comparison of average downstream rate vs. average PSNR per user profile and remote desktop protocol.

Fig 16 displays the average PSNR and average downstream bitrate for each remote desktop protocol and user profile. The downstream rate is in a logarithmic scale to accommodate the wide range of values.

In the lower left corner, TeamViewer presents the lowest PSNR; however, it also consumes the least amount of bandwidth. Other protocols requiring less than 1 Mb/s do not sustain this rate for all user profiles. For example, RDP requires less than 1 Mb/s for the office and web browsing user profiles, yet requires an average of 3 Mb/s for the video user.

TeamViewer simplifies link bandwidth dimensioning when measuring only average bit rates. However, different user profiles present significant differences in the traffic long-range dependence, which influence packet buffer dimensioning. TeamViewer is an extreme case of this situation as it indicates a Hurst parameter as low as 0.693 for an office user and as high as 0.946 for a web user (Table 5). It does this at the expense of important losses on quality for highly dynamic desktops, where it remains at a PSNR less than 25 dB (an MOS in the “Poor” or “Annoying” range).

VNC requires a large link capacity for any dynamic content (the web browsing and video profiles), obtaining low QoE owing to the delays in rendering. It is a reasonable solution only for an office user with infrequent changes of large parts of their screen.

PCoIP maintains an acceptable quality (MOS at least “3”) for every user profile, with a reasonable link capacity requirement for the office and web browsing profiles. The video case requires several megabits per second, however it offers an increased quality compared to every other desktop system.

For the office user profile, the best quality at the least cost is obtained by the protocols that transfer system graphics commands (ICA and RDP). This is true both on bit rates and on values of H. They do not require sending screen bitmap updates; rather, they send the instructions to recreate the GUI status at the client (opening a window, placing text using a local font). This typically requires smaller downstream updates and shorter bursts. For video playback and some video content in web browsers, these systems transfer the video file for local playback using an independent communications channel, obtaining acceptable quality with a reasonable link capacity, as the original compressed file they transfer is typically smaller than the result of the on-the-fly compression of the screen updates.

Based on the MOS scale, certain combinations of user profile and RD system should be excluded. VNC is not suitable for a video user and TeamViewer does not provide sufficient quality for video and web browsing with highly dynamic content. For an office user, TeamViewer does not provide sufficient quality. Other solutions with a similar bitrate provide a superior experience.

For a web browsing user, RDP and ICA likely offer the best trade-off between bitrate and quality. PCoIP must compress the animations in the web page as a video stream and therefore obtains lower quality, even with higher bitrates.

For a video user, RDP, ICA, and PCoIP present acceptable quality, with the lowest bitrate achieved by the RDP solution. Between ICA and PCoIP, the latter offers improved quality at the same cost.

Optimum link capacity cannot be determined based only on the average expected traffic. The self-similar nature of remote desktop traffic is clear and it is not alleviated with reasonable degrees of traffic multiplexing. For a mixture of users, the worst profile (the web profile) dominates in the resulting traffic. Depending on the number of users and the number from each profile in the traffic mix, the result will be closer to the behaviour of the strongest long-range dependent traffic.

## Recommendations for remote desktop protocol design

The most important suggestions that can be extracted to improve user experience in DaaS solutions are:

- Protocols that transfer system graphics commands (ICA and RDP) are better suited to office user profiles because functions such as the frequent opening and closing of system windows, menu scrolling, and text inputs are not transferred as screen image updates through the network. They avoid streaming the user screen as video, as they transmit system graphics commands. This means lower traffic bit rates with high image quality, achieving low response times, and therefore the best QoE.
- Protocols that transfer system graphics commands (ICA and RDP) also achieve acceptable results in web browsing and video profiles because they use specific channels to transfer the content (H.264 video, Adobe flash, audio, DirectX). Each content is coded according to its nature and, if possible, is transmitted without further compression, using the original source data that is already compressed and adapted to be streamed over the network (for example, a YouTube video). RDP and ICA offer the best trade-off between bitrate and quality. However, the client PCs must be more powerful (computationally speaking) because they must process content from the specific channels, sometimes using complex codecs.
- Multiplexing hundreds of users with an office profile provides less long-range dependence (lower Hurst parameter) for ICA and RDP, as they use system graphics commands instead of streaming a video from the full screen as in other protocols. Even with the web and video profiles, the resulting H value for multiplexed users is better than for the other protocols. This means that the required bandwidth in the Internet link will increase smoothly with the number of simultaneous users.
- In some protocols (PCoIP, VNC, TeamViewer), all content is streamed as screen bitmap updates. Therefore, the differences among office, web, and video profiles are related to the size and speed of the changes in the screen images. In this case, the web profile has, surprisingly, the highest H value and larger link data rate requirements than the video profile for the same MOS. This is because of the full screen updates required when scrolling a web page or the embedded advertisements.
- Protocols can offer low bit rates using complex codecs with lossy compression (TeamViewer is an example). However, they accomplish this at the expense of a reduced MOS and in some situations, they result in a greater degree of self-similarity in the traffic. This makes link capacity dimensioning more complex and packet buffers less effective to reduce losses, as the traffic contains larger bursts.

## Conclusions

We compared five of the most popular remote desktop protocols and offered models for their traffic arrival based on self-similar processes. They were deployed in a public virtual cloud as a DaaS solution. The protocols were: PCoIP as used in the Amazon WorkSpaces, Microsoft RDP, TeamViewer, VNC (RFB), and Citrix ICA. We evaluated the network transfer rate and its relation to the quality experienced by the DaaS user. We compared three different user behaviours based on productivity: an office software suite, web browsing to modern and dynamic websites, and a video user accessing low and high quality video streams.

The QoE measurement was accomplished by comparing the desktop video stream at the source (the server in the cloud) and the destination (the user client). An objective PSNR time series was obtained from the comparison and from this, we produced subjective MOS values. This evaluation considered not only image degradation due to lossy compression but also loss of interactivity from an increased delay, as resulted in video stream desynchronization.

The results demonstrate that the Amazon WorkSpaces solution (based on PCoIP) presents a reasonable quality for the three user profiles, although its network bandwidth usage for a video user is considerably greater than the recommended values suggested by Amazon. We confirmed that the recommended traffic rates of 300–1000 Kb/s are reasonable for the office profile. However, for the web browsing and video profiles, we determined that sustained rates up to 10 Mb/s are common. Moreover, the degree of self-similarity in network traffic is greater for web users than for the other user profiles, including video consumers. A network administrator must consider this when dimensioning an access link for a population of Amazon WorkSpaces users.

Protocols based on the transfer of graphics primitives (such as RDP and ICA) offer high quality with a low traffic bit rate for a normal productivity desktop user. For multimedia playback, they include parallel channels for the transfer of video files instead of streaming an on-the-fly compressed video extracted from the screen.

Solutions such as VNC and TeamViewer are less suited for a DaaS deployment and a better fit for remote control of physical desktops during short tasks. TeamViewer primes a low network bandwidth usage at the expense of the quality, hence it is an acceptable solution in remote assistance scenarios where the interaction is short and high quality is not required. VNC is the simplest system; hence, it offers minimal optimisation compared to the other analysed solutions. The result is high traffic bitrates and less than proportional quality as the compression task introduces delays that degrade the interactivity.
